# Treatment response to bupropion: an investigation of changes in resting-state functional connectivity in patients with major depressive disorder

**DOI:** 10.1007/s00213-025-06993-7

**Published:** 2025-12-23

**Authors:** Sandra E. Klonteig, Annabel E. L. Walsh, Rune Jonassen, Michael Browning, Catherine J. Harmer, Marieke A. G. Martens

**Affiliations:** 1 Faculty of Technology, Art, and Design, Oslo Metropolitan University, Pilestredet 46, Postrommet, 0167 Oslo, Norway; 2 Department of Health Research, Sintef Digital, Oslo, Norway; 3 The McPin Foundation, London, UK; 4 Faculty of Health Sciences, Oslo Metropolitan University, Oslo, Norway; 5 Department of Psychiatry, University of Oxford, Oxford, UK; 6 Oxford Health NHS Foundation Trust, Oxford, UK

**Keywords:** Depression, Bupropion, Resting-state fMRI, Resting-state networks, Treatment response, Independent component analysis

## Abstract

**Rationale:**

Bupropion’s unique pharmacology as a dopaminergic and noradrenergic reuptake inhibitor distinguishes it from other antidepressants. Studies on a number of other antidepressants have demonstrated modulation of resting-state functional connectivity (rsFC). However, how bupropion affects rsFC in patients with major depressive disorder (MDD) remains unknown.

**Objectives:**

To identify rsFC changes in MDD patients receiving bupropion treatment relative to a sample of controls.

**Method:**

Thirty-nine MDD patients received 300 mg (titrated from 150 mg) Bupropion daily for 6 weeks. 40 controls received no treatment. All participants underwent MRI scans at baseline and after 2 weeks. Mood and symptom severity were assessed at baseline, week 2, and week 6. Comparisons of rsFC were made at baseline and for *changes* from baseline to the 2-week follow-up. Correlations between symptoms and rsFC were also assessed.

**Results:**

In this explorative study, no significant baseline differences were found between patients and controls. Crossover interaction effects between groups and time were observed. Network analysis identified *changed* rsFC between the cerebellar network and left superior and middle temporal gyrus. Seed analysis showed changed rsFC between the insula and areas in the sensorimotor and occipital cortex as well as precuneus. No relationships were found between changes in symptoms at week 6 and rsFC changes at week 2.

**Conclusions:**

This study is the first to examine bupropion’s effects on rsFC in MDD patients, revealing rsFC changes that may be important for bupropion’s mode of action. These preliminary findings provide a foundation for further investigation into bupropion and its underlying mechanisms, which may have important implications for depression treatment.

**Supplementary Information:**

The online version contains supplementary material available at https://doi.org/10.1007/s00213-025-06993-7.

## Introduction

Major depressive disorder (MDD) affects over 185 million people worldwide (Mental Disorders Collaborators 2022). The most common first-line pharmacological treatments for MDD are selective serotonin reuptake inhibitors (SSRIs) and noradrenergic antidepressants (SNRIs) (Bauer et al. 2013). However, only about one-third of patients respond to their first treatments with antidepressants, and it may take up to several months to find an effective therapy (Rush et al. 2009). Given this lack of efficacy and the known heterogeneous nature of MDD, further research is needed on *atypical* antidepressants that might prove more effective for certain individuals and specific MDD symptoms (Zarate et al. 2013).

Bupropion is one such antidepressant, typically offered as a fourth-line treatment (Patel et al. 2016). It is distinguished from other antidepressants by its unique pharmacology as a dopaminergic and noradrenergic reuptake inhibitor without prominent serotonergic effects (Ascher et al. 1995). Due to its actions on the dopamine system, it may be particularly effective in treating anhedonia, a core symptom of MDD characterized by the loss of pleasure or interest in previously enjoyable activities (American Psychiatric Association 1994; Cao et al. 2019; Nutt et al. 2007; Patel et al. 2016; Tomarken et al. 2004). A large body of literature links dopamine to brain regions involved in reward processing, such as the striatum, prefrontal cortex, and insular cortex (Haber and Knutson 2010). However, the precise neural mechanisms underlying bupropion’s beneficial effects and their implications for the treatment of MDD are not fully understood.

Network approaches are promising and frequently used methods for researching MDD and the potential mechanisms of antidepressants (Dichter et al. 2015). This is in line with MDD being conceptualized as a *network disorder*, meaning it is not necessarily linked to abnormalities in individual brain regions but rather to the connections and interactions between these regions (Li et al. 2018). A common method for studying this hypothesis is the measurement of the temporal correlation of activity between brain regions, known as functional connectivity (FC), using functional magnetic resonance imaging (fMRI). Resting-state functional connectivity (rsFC) refers to the measurement of FC while someone is awake and not engaged in any activity or task. An advantage of resting state–based approaches is that they are non-reliant on task performance, also offering the important benefit of being reproducible across different populations and study settings.

The default mode network (DMN), the executive control network (ECN), and the salience network (SN) are well-documented resting-state networks (RSNs) that are important for the regulatory functions involved in various cognitive and emotional processes (Menon 2011). The triple network model proposes that dysfunctional connectivity within and between these networks—DMN (important for self-referential processing), ECN (cognitive control), and SN (stimulus detection)—is linked to MDD (Menon 2011). For example, MDD has been associated with aberrant connectivity within SN, between DMN and SN, the frontal areas of ECN, and between the frontal nodes of ECN and SN, which has been interpreted as inadequate top-down regulation of attention and emotion (Williams 2017; Zhong et al. 2016).

In line with this, studies on antidepressants, including SSRIs, SNRIs, and the norepinephrine reuptake inhibitor reboxetine, have shown modulation of connectivity between these networks (Brakowski et al. 2017; Dichter et al. 2015; Martens et al. 2022; Vai et al. 2016). For example, it has been found that a better antidepressant response is associated with increased connectivity between the frontal and limbic regions, and between the frontal and DMN regions (Dichter et al. 2015). These findings are not extensively documented for bupropion, but a study by Rzepa et al. (2017) showed that 7 days of bupropion treatment, relative to placebo, increased rsFC in central parts of the DMN, namely, the dorsal medial prefrontal cortex and the precuneus. However, this study was based on a healthy population and, to our knowledge, has not been studied in a clinical sample.

The present study is the first to investigate how treatment with bupropion alters rsFC in patients with MDD. Therefore, we employed a broad, data-driven approach in our primary analyses using independent component analysis (ICA). This method also allows for the discovery of new regions potentially affected by bupropion, from which new hypotheses can be formulated (Yang et al. 2020). Furthermore, in our supplementary analyses, we conducted hypothesis-driven seed-based analyses to explore changes in specific regions associated with DMN, SN, and ECN, including the nucleus accumbens, caudate nucleus, putamen, insula, medial prefrontal cortex/anterior cingulate cortex, and precuneus. Our research questions and hypotheses were as follows:

1. Are there baseline rsFC differences in MDD patients compared to controls? This comparison was conducted to identify rsFC patterns that were unique to MDD patients. Based on previous studies, we expected baseline differences between controls and patients in brain regions related to the DMN, SN and ECN.
2. Is there a significant effect of bupropion treatment on rsFC networks in MDD patients? We hypothesized significant *changes* in the MDD group from baseline to follow-up, compared to changes in controls. This is based on studies with other types of antidepressants, showing that these may alter rsFC in relevant networks.
3. Do bupropion-related rsFC changes correlate with changes in symptom severity after 2 and 6 weeks? We hypothesized that these changes correlate with reduced symptoms, with potentially stronger correlations with symptoms of anhedonia.

## Method

Data were collected as part of a larger study at the University of Oxford from January 2014 to April 2016 (Walsh 2016), which was approved by the University of Oxford Clinical Trials and Research Governance Team and the National Health Service Research Ethics Committee (NRES Committee South Sentral – Berkshire B) (13/SC/0569). The study was carried out in accordance with the World Medical Association’s (2013) Declaration of Helsinki, and all participants signed a written consent form. The study was not preregistered.

### Participants

A total of 80 participants, both male and female, aged between 18 and 50 years, were recruited for the study. This number included 40 patients diagnosed with MDD. An equal number of controls were recruited to provide a matched comparison, ensuring that the groups were balanced in terms of age, gender, and verbal IQ (derived from the National Adult Reading Test; Nelson and Wilison 1991). Various channels were used for recruiting, including internet postings, public space advertisements, and radio broadcasts. The inclusion criterion for MDD participants was a diagnosis of a current MDD episode, as determined by the Structured Clinical Interview for DSM Disorders. The study did not include MDD patients with other psychiatric disorders, except for those with comorbid anxiety disorders. The general exclusion criteria for all participants included having undergone psychological treatment within the last 3 months, recent usage of psychotropic drugs or medications, heavy smoking habits (10 cigarettes daily or greater), and any contraindications to MRI scanning.

### Procedures and materials

All participants were assessed through a screening visit that evaluated their medical histories, drug dependencies, IQ, medication use, and pregnancy status (if applicable). Urine drug tests were also performed. Participants with MDD underwent a more comprehensive psychiatric evaluation, which included an investigation into their histories with MDD and physiological measurements, such as blood samples, heart rate, and ECG.

The participants then attended a baseline session (test visit 1), which included measurements of mood and symptom severity, emotional and cognitive processing, and rsFC. The following day, the participants in the MDD group began a 6-week treatment with open-label SR Bupropion, starting with 150 mg and then increasing to 300 mg after a clinical review between days 7 and 10. Note that the study did not include a placebo control group due to ethical concerns about leaving the patient group untreated for a long period with a placebo instead of true medication. To allow assessment of repeat testing, the controls were not given any medication, placebo, or other intervention during this six-week period. All participants repeated measurements from the baseline session after 2 (test visit 2) and 6 weeks (test visit 3). However, at the six-week follow-up, they did not undergo any MRI scans to limit participant burden and cost. The 2-week and 6-week time points were chosen based on the duration for bupropion to reach steady state (Jefferson et al. 2005), as well as the duration for patients to clinically respond to bupropion (Patel et al. 2016).

The materials relevant to this study included questionnaires on mood and symptom severity. Specifically, these were the Hamilton Rating Scale for Depression (HAM-D), the Full Mood and Anxiety Symptom Questionnaire (MASQ), and the Snaith–Hamilton Pleasure Scale (SHAPS). In addition, the side effects of antidepressants were measured with the Oxford Questionnaire on the Emotional Side Effects of Antidepressants. These were previously reported by Walsh et al. (2018), and the present study reports on all participants with complete resting-state data at both time points. The MRI scans included structural imaging and functional activity recording during behavioral tasks and rest periods. During the resting-state fMRI session, the participants were instructed to stay still, keep their eyes open, look at a white fixation cross presented on a black background, blink normally, think of nothing in particular, and not fall asleep.

### Data acquisition

All imaging data were acquired on a 3.0 Tesla TIM Trio scanner. A total of 180 volumes of T*2-weighted echo planar images (EPIs) were obtained using a voxel resolution of 3 × 3 × 3 mm^3^, repetition time (TR) = 2.71 s, echo time (TE) = 35 ms, flip angle (FA) = 87°, slice angle = 30°, and duration time = 6 min. T1-weighted structural images were acquired using a magnetization prepared rapid acquisition by gradient echo sequence (MPRAGE) with a voxel resolution of 1 × 1 × 1 mm^3^, TR = 2.4 s, and TE = 4.7 ms.

### Preprocessing of resting-state fMRI data

The SynthStrip tool was used to extract brain from nonbrain material in the high-resolution structural data (Hoopes et al. 2022; Kelley et al. 2024). Resting-state fMRI data were analyzed using the Functional Magnetic Resonance Imaging of Brain (FMRIB) Software Library (FSL version 6.0.7.13, FMRIB analysis group, Oxford University, UK) (Jenkinson et al. 2012).

Single-subject functional MRI data were preprocessed and analyzed using Multivariate Exploratory Linear Decomposition into Independent Components (MELODIC). Preprocessing involved several steps designed to reduce unwanted variability in the data and to improve the validity of the statistical analysis. The following steps were implemented for each subject: (1) The prestatistics procedures included motion correction with MCFLIRT (Jenkinson et al. 2002), brain extraction with BET (Smith 2002), spatial smoothing with a Gaussian kernel of FWHM 5 mm, grand-mean intensity normalization, and high-pass temporal filtering with Gaussian-weighted least-squares straight line fitting with sigma set to 75 s. Resting state–specific preprocessing steps included masking of nonbrain voxels, voxel-wise data de-meaning, and normalization of voxel-wise variance. Data were registered to structural space with FLIRT (Jenkinson et al. 2002) and to MNI standard space with FNIRT nonlinear regression with warp resolution = 10 mm and resampling resolution = 2 mm (Andersson et al. 2007).

Resting-state analysis was carried out using probabilistic independent component analysis (Beckmann and Smith 2004), as implemented in MELODIC Version 3.15, which is part of FSL (FMRIB’s Software Library, www.fmrib.ox.ac.uk/fsl). The preprocessed data were whitened and projected into a 72-dimensional subspace using probabilistic principal component analysis, in which the number of dimensions was estimated using Laplace approximation to the Bayesian evidence of the model order (Minka 2000).

The whitened observations were decomposed into sets of vectors that described signal variation across the temporal domain (time courses) and across the spatial domain (maps) by optimizing for non-Gaussian spatial source distributions using a fixed-point iteration technique (Hyvärinen 1999). The estimated component maps were divided by the standard deviation of the residual noise and thresholded by fitting a mixture model to the histogram of intensity values (Beckmann and Smith 2004).

Resting-state data were then denoised by independent component analysis (ICA) using FMRIB’s ICA-based Xnoiseifier (FIX) by manually creating a training dataset from five subjects (randomly chosen) in each group and each test visit (*N* = 20). Manual labeling of the ICA components as noise or signals was completed independently by two researchers (SK and MM). Their correspondence was 90% and 95% for test visits 1 and 2, respectively. In the event of disagreements, the components were revisited for a final determination.

### Network ICA analysis

The preprocessed, cleaned functional data were then temporally concatenated across subjects to create a single 4D dataset. The (group-wise) concatenated multiple rs-fMRI datasets were then decomposed using group ICA to identify large-scale rsFC patterns in the population of subjects. The ICA was constrained to 15 components, as this split provided the most optimal results for our dataset. This decision was a trade-off between splitting components up into smaller constituents (like the DMN) and merging other components of interest (like ECN and left/right frontoparietal network). Components were either identified to reflect movement or scanner or physiological noise or as corresponding to known RSNs. This was done by visual inspection independently by MM and SK, and additionally through comparisons to previously published maps (Smith et al. 2009) using Pearson spatial cross-correlation. After resting-state components were identified, subject-specific versions of the spatial maps and associated time series were generated based on the set of spatial maps from the group-average analysis using dual regression (Beckmann et al. 2009). Specifically, for each subject, the group-average spatial maps were first used as spatial regressors in a multiple regression analysis applied to the subject’s 4D space–time dataset. This process produced a set of subject-specific time series corresponding to each group-level spatial map. Subsequently, these time series were employed as temporal regressors in another multiple regression analysis on the same 4D dataset, yielding a set of subject-specific spatial maps, each aligned with a group-level spatial map. Subject-specific spatial maps were then combined with fslmerge in order of the design matrix to generate the relevant input for randomise (see group-level statistical analysis).

### Seed analysis

To incorporate a hypothesis-driven approach, we also performed a seed-based analysis. The seed-based approach involved several steps to analyze the data. Seed masks were first created and registered in functional space. We followed the methodology outlined by Yarkoni et al. (2011) to create the DMN seed masks. This included the use of NeuroSynth, which generates meta-analytic whole-brain maps through automated text-based data mining of abstracts from published neuroimaging studies. Specifically, we used the search term “Default Mode,” generating an automated meta-analysis of 700 studies (www.neurosynth.org) (December 22 2024). Using this map, we identified coordinates with a peak z-score within the anterior (x = − 2, y = 50, z = − 6) and posterior (x = 2, y = − 54, z = 26) DMN using the FSL tool FSLmaths. To ensure regional specificity, we created masks by overlapping the NeuroSynth map with 10 mm-radius spherical masks centered on the peak coordinates. Midbrain striatum seeds were also included, as these regions are central to the dopaminergic system. Bilateral caudate, putamen, and nucleus accumbens masks were combined based on the Harvard–Oxford Atlas included within FSL (masks thresholded at 50). Next, the time-series data for each mask were extracted. First-level connectivity was calculated as the correlation (both positive and negative) of time series of the seed with all other voxels in the brain using FSL FEAT. A white matter and cerebral spinal fluid (CSF) mask were created in standard space and then registered into the individual’s functional space before being included as covariates of no interest. Contrast of parameter estimates (copes) were then registered to standard space and combined to generate input for randomise.

### Group-level statistical analysis

We analyzed differences between controls and patients using FSL’s randomise permutation-testing tool with 5,000 permutations and the threshold-free cluster enhancement method, with a family-wise error-corrected cluster significance threshold of *p* <.05 applied to suprathreshold clusters (Nichols and Holmes 2002; Smith and Nichols 2009). Only significant clusters larger than 10 voxels located in gray matter were further interpreted. Other results were not interpreted but were included in the reported results. Nonsignificant findings are reported in the supplementary material for completeness and for future meta-analyses. To further visualize the results, we extracted individual parameter estimate (PE) values from their custom maps using significant clusters as binary masks.

The following analyses were conducted for both the network ICA and seed analysis:

1. *Baseline comparison*: A two-sample group comparison (controls vs. patients) of rsFC at baseline was conducted.
2. *Comparison of changes in rsFC*: A two-sample group comparison (controls vs. patients) of *changed* rsFC was performed. Change was calculated by manually computing the differences between visits (test visit 2 − baseline) for each subject using FSLmaths., A one-sample t-test comparison of rsFC difference scores (test visit 2 − baseline) was also performed for the MDD group only as a sensitivity analysis.
3. *Correlations between change in* symptom severity *and rsFC*: Additionally, we performed Pearson’s R correlations between *change* in the identified rsFC measures (PEs) and change in symptom severity. Specifically, we calculated *change* in symptom severity from baseline to 2 weeks (test visit 2 − baseline) and until the end of treatment (test visit 3 − baseline) for the HAM-D, Full-MASQ, and SHAPS; *change* in rsFC was calculated as test visit 2 − baseline.

## Results

### Clinical and demographic data

The study included 46 MDD patients and 42 controls. Four participants in the patient group were excluded due to MRI-related issues/artifacts. Five participants (3 patients, 2 controls) dropped out of the study and did not attend the follow-up visit, leaving a total sample of 39 MDD patients and 40 controls. See Table 1 for the final sample characteristics at baseline, and Table 2 for mood and symptom severity changes over time (from baseline to test visit 3 at 6 weeks). The effects in this subsample are similar to those reported by Walsh et al. (2018).

**Table 1:** Sample characteristics at baseline

	Patients	Controls
*n*	39	40
Gender (male: female)	10:29	13:27
Age	30 (8)	29 (9)
HAM-D	13.2 (3)	0.9 (1)
SHAPS	34 (6)	20 (4)
MASQ		
Anhedonic depression	83 (10)	45 (7)
General distress: anxious	26 (7)	14 (4)
General distress: depressive	40 (9)	16 (5)
General distress: mixed	46 (9)	22 (5)
Anxious arousal	28 (8)	19 (4)
Length of current episode (months)	46.7 (56.0)	
Number of previous episodes	3.8 (7.0)	
Age of onset (years)	17.4 (7.3)	
Previous treatment	Yes: 31 No: 8	

Values are reported as means (standard deviations [SDs]). *p*-value from Wilcoxon rank sum test. HAM-D: Hamilton Depression Rating Scale; MASQ: Mood and Anxiety Symptoms Questionnaire

**Table 2:** Mood and symptom severity at baseline and changes over time

	Patients					Controls			
Questionnaire	v1		v2	v3	Change	v1	v2	v3	Change
*n*	39			38		40	40	40	
HAM-D	13 (3)		11 (3)	6 (3)	< 0.001*	1 (1)	1 (1)	1 (2)	0.97
SHAPS	34 (6)		31 (5)	27(7)	< 0.001*	20 (4)	20 (4)	20 (5)	0.76
MASQ									
Anhedonic depression	83 (10)		74 (15)	63 (16)	< 0.001*	45 (7)	44 (9)	45 (14)	0.71
General distress: anxious	26 (7)		22 (7)	20 (6)	< 0.001*	14 (4)	14 (3)	13 (3)	0.29
General distress: depressive	40 (9)		33 (11)	25 (8)	< 0.001*	16 (5)	15 (5)	16 (7)	0.58
General distress: mixed	46 (9)		41 (10)	34 (9)	< 0.001*	22 (5)	21 (5)	21 (6)	0.64
Anxious arousal	28 (8)	27 (8)		26 (7)	0.28	19 (4)	18 (1)	18 (1)	0.03*

Values are reported as means (SDs). v1, 2, and 3; Test visits 1, 2, and 3. HAM-D: Hamilton Depression Rating Scale; MASQ: Mood and Anxiety Symptoms Questionnaire. Change is calculated using one-way repeated measures ANOVA, reporting the p-value. * indicates a *p*-value < .05

### Network ICA analysis

**Fig. 1:**
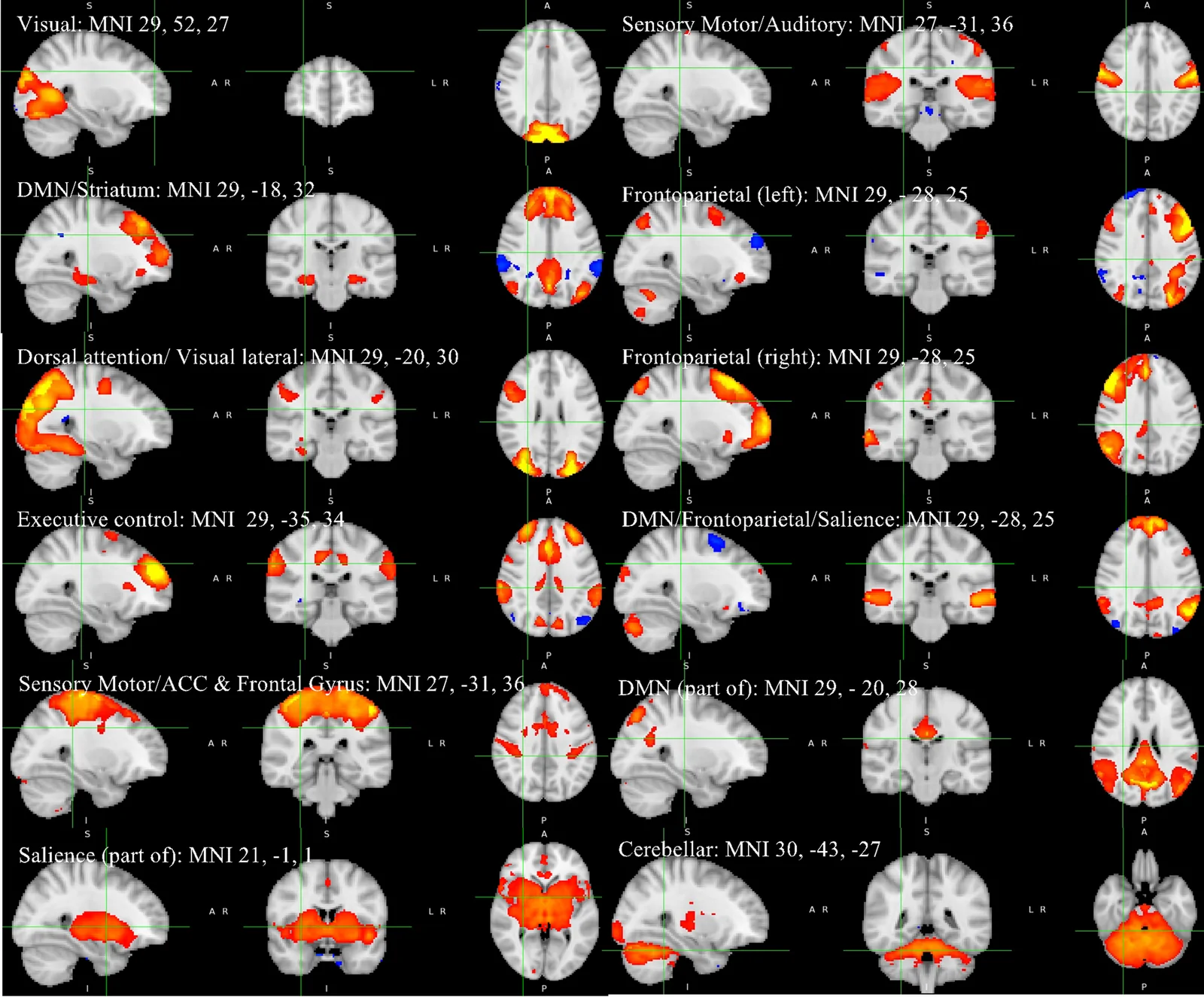
Resting-State Networks Identified in the Study. *Note.* Axial, coronal, and sagittal views for the main rsFC networks, overlaid onto the standard MNI brain. The cerebellar network is not complete due to the field of view of the functional scan

From the 15 independent components extracted through temporal concatenation, 12 components were identified as RSNs of interest, corresponding to previously recognized RSNs (Smith et al. 2009; Yeo et al. 2011). The remaining components were classified as noise that was not eliminated by the artifact rejection algorithm (FIX), encompassing artifacts from physiological noise (e.g., arteries, veins, and cerebrospinal fluid), head motion, and scanner artifacts. All RSNs of interest are illustrated in Fig. 1.

**Fig. 2:**
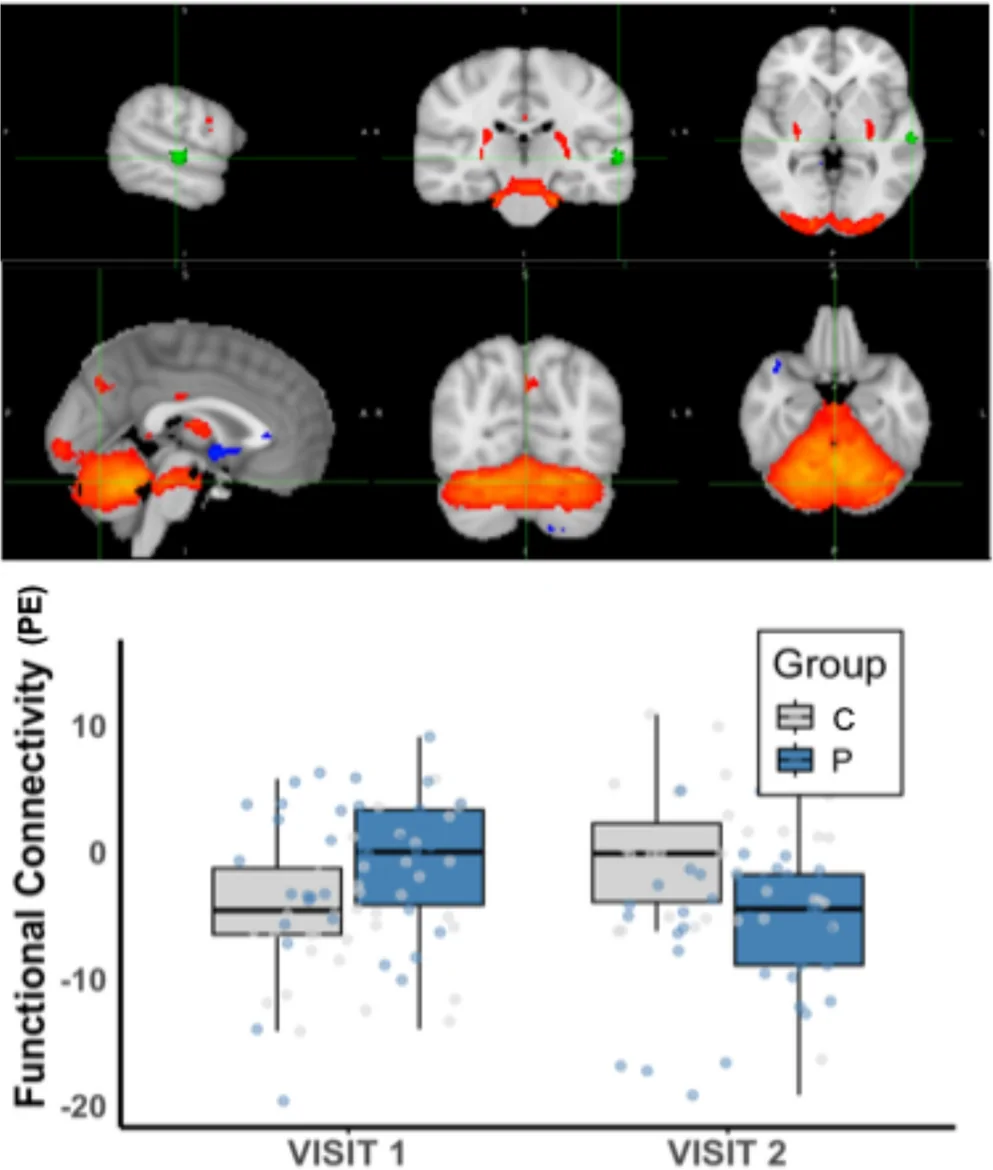
Significant Differences in Functional Connections between Resting-State Networks and Individual Brain Regions in Patients and Controls. *Note.* Top (MNI − 60,−24,0): Change (2 weeks − baseline) in rsFC in controls compared with patients between the cerebellar network and the posterior and middle divisions of the left superior temporal gyrus. Green: Group difference between patients and controls. Red-yellow: Cerebellar network, regions with a correlated BOLD signal time course. Blue: cerebellar network, regions with an anti-correlated BOLD signal time course. Middle (MNI 30, −43, −27): Axial, coronal, and sagittal views of the cerebellar network, overlaid onto the standard MNI brain. The cerebellar network is not complete due to the field of view of the functional scan. Bottom: Graphs showing boxplots with parameter estimates (beta values) extracted from baseline and test visit 2 for P and C for each network. The horizontal line inside each box represents the median, while the whiskers extend to the farthest data point within 1.5 times the interquartile range from the quartiles. P: Patients; C: Controls, PE: Parameter Estimates

Nonparametric permutation analysis revealed the following:

1. *Baseline comparison*: No significant differences between patients and controls were found at baseline. See Table S1.
2. *Comparison of changes in FC*: Results showed significant group differences in changed rsFC between controls and patients in certain networks. Specifically, patients exhibited less change in rsFC between the cerebellar network and the superior and medial temporal cortex compared to controls. Exploring directionality showed that rsFC increased over time for the controls, while it decreased for the patients. There were also differences in the frontoparietal network and dorsal attention/visual lateral network, although these were mostly elated to white matter regions and only in a small number of voxels (< 10), respectively. See Table 3; Fig. 2. Nonsignificant differences are presented in Table S3. The sensitivity analysis revealed no significant changes in rsFC for patients only (see Table S3).
3. *Correlations between changes in symptom severity and FC*: There were no significant correlations between change in rsFC and change in HAM-D, MASQ, or SHAPS scores (at week 2 or 6), with the correlation coefficients ranging from *r* = −.18 to 0.17 and with p-values ranging from 0.29 to 0.74.

### Seed analysis

**Fig. 3:**
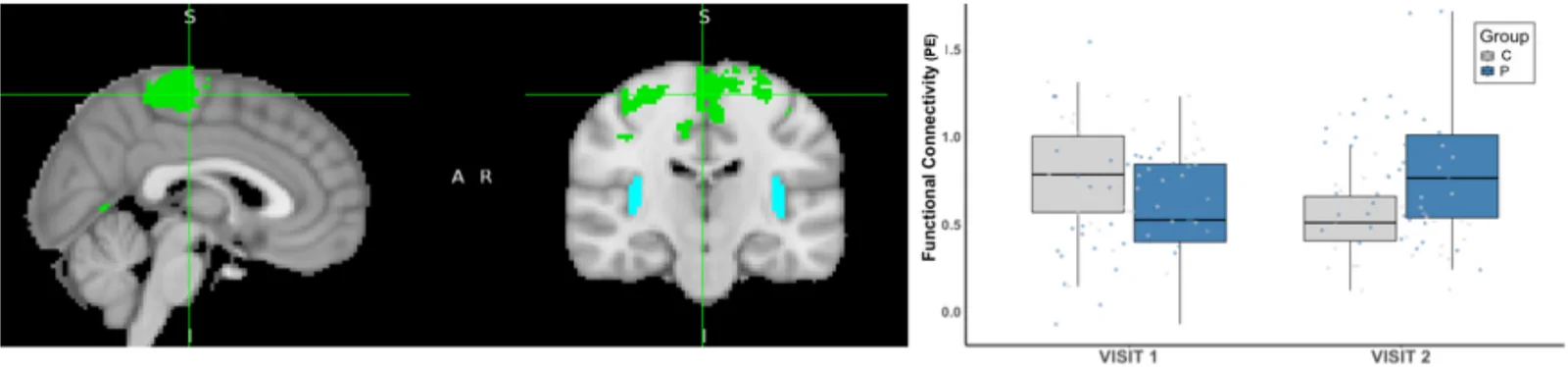
Significant Differences in Functional Connections between Insula Seed and Individual Brain Regions in Patients and Controls. *Note.* Left (MNI 2, −20, 62): Change (test visit 2 − baseline) in FC in P compared with C between the insula seed (blue) and bilateral precentral gyrus, extending into the postcentral gyrus, posterior cingulate gyrus, supplementary motor cortex, superior frontal gyrus, right inferior frontal gyrus, right middle frontal gyrus, right central opercular cortex, left superior parietal lobule, and left lateral occipital cortex (green). Right: Graphs show a boxplot with PEs (beta values) extracted from the significant clusters. The horizontal line inside each box represents the median, while the whiskers extend to the farthest data point within 1.5 times the interquartile range from the quartiles. P: Patients; C: Controls

1. *Baseline comparison*: No significant differences between patients and controls were found at baseline. See Table S2.
2. *Comparison of changes in FC*:Seed-based analyses revealed significantly greater changes in rsFC in patients compared to controls between the insula and a range of regions, including the sensorimotor (e.g. precentral and postcentral gyrus) and occipital cortex (e.g. occipital pole and fusiform gyrus), as well as the precuneus (see Table 4; Fig. 3). Exploring directionality showed that rsFC decreased over time for controls, whilst it decreased for the patients. For nonsignificant differences, see Table S4. The sensitivity analysis revealed no significant changes in rsFC for patients only (see Table S4).
3. *Correlations between changes in symptom severity and FC*: No significant correlations were found between change in rsFC and change in HAM-D, MASQ, or SHAPS scores (at week 2 or 6), with the correlation coefficients ranging from *r* = −.16 to 0.08 and with *p*-values ranging from 0.34 to 0.96.

**Table 3:** Significant changes (2 weeks – baseline) in functional connections between Resting-State networks and individual brain regions in patients and controls

Contrast	Network	Cluster	Cluster size (number of voxels)	Peak voxel	*p*-value
P > C	Dorsal attention/visual lateral	Right lateral occipital cortex, extending to the occipital fusiform gyrus	2	44, − 80, −12	0.045
C > P	Left frontoparietal	Right insular cortex, extending to the frontal orbital cortex, note mostly white matter	73	28, 26, 4	0.006
C > P	Cerebellar	3: Left superior and middle temporal gyrus extending to the planum temporale	78	−60, − 24, 0	0.014
		2: Left temporal pole	2	−56, 6, −6	0.05
		1: Left temporal pole	1	−54, 10, −8	0.049

Significant changes (test visit 2 − baseline) functional connections (temporal correlations/cohesion) between RSNs and individual brain regions, with individual clusters identified within each contrast. P: Patients; C: Controls

**Table 4:** Significant changes in functional connections between the Insula seed region and individual brain regions in patients and controls

Contrast	Cluster	Cluster size (Number of voxels)	Peak voxel	*p*-value
P > C	Bilateral precentral gyrus, extending into the postcentral gyrus, posterior cingulate gyrus, supplementary motor cortex, superior frontal gyrus, right inferior frontal gyrus, right middle frontal gyrus, right central opercular cortex, left superior parietal lobule, and left lateral occipital cortex	4,399	2, − 26, 62	0.001
	Right lateral occipital cortex extending into the occipital fusiform gyrus and occipital pole	1173	34, − 80, 24	0.021
	Left lateral occipital cortex, extending into the occipital fusiform gyrus and occipital pole	701	−28, − 86, 2	0.026
	Left precentral gyrus	241	−46, − 8, 60	0.04
	Right lateral occipital cortex, precuneus, superior parietal lobule	181	20, − 58, 58	0.038
	Left superior frontal gyrus	32	−14, 2, 66	0.044
	Left precuneus cortex, lateral occipital cortex	22	−4, − 64, 60	0.046
	Left lingual gyrus	17	0, − 62, 4	0.045
	Right lingual gyrus, extending into the occipital fusiform gyrus	13	16, − 86, −6	0.049
	Right temporal occipital fusiform cortex	11	46, − 58, −18	0.048
	Left supplementary motor cortex	11	−4, 0, 58	0.049
	Left lateral occipital/middle temporal gyrus	7	−40, − 58, 4	0.047
	Lateral occipital cortex, occipital pole	1	−18, − 88, 28	0.05

Significant changes (test visit 2 − baseline) and functional connections (positive temporal correlations) between seed regions and individual brain regions, with individual clusters identified within each contrast. P: Patients; C: Controls; ACC: Anterior Cingulate Cortex

## Discussion

The main aim of this study was to investigate the effects of 14 days of bupropion treatment on rsFC in patients with MDD compared to controls undergoing no treatment. While we did not observe significant baseline differences between the two groups, we found that patients had significantly less *change* in rsFC over time (compared to controls) between the cerebellar network and superior/middle temporal gyrus in a network ICA analysis. Exploring directionality revealed that rsFC increased over time for controls, while it decreased for patients. In the seed analysis, the patients had greater change in rsFC between the insula seed and areas in the sensorimotor and occipital cortex, as well as in the precuneus, compared to the controls. Here, rsFC increased over time for the patients, while it decreased for the controls. Therefore, crossover interaction effects between group and time were observed. No significant effects of bupropion were found when assessing change for the patients only. Moreover, no relationship was found between changed severity of symptoms and changed rsFC.

Our analyses revealed no significant baseline differences in rsFC between controls and MDD patients, which is not in line with previous studies linking MDD to alterations in DMN, SN, and ECN (Menon 2011). For example, a meta-analysis by Kaiser et al. (2015) found *hyper*connectivity within DMN and between DMN and frontoparietal control systems in MDD, while areas in SN and ECN showed *hypo*connectivity. Similarly, in a mega-analysis of 606 MDD patients and 476 controls, Javaheripour et al. (2021) found *hypo*connectivity in regions related to SN and ECN, but no significant differences in the DMN or frontoparietal networks. More recently, Zhang et al. (2025) replicated some of these findings. In their meta-analysis they found both increased and decreased connectivity within the DMN and increased connectivity within the FPN. Between-network findings moreover showed increased DMN–FPN and limbic network (LN)–DMN connectivity, decreased DMN–somatomotor network and LN–FPN connectivity, and varied ventral attention network (VAN)–dorsal attentional network (DAN) connectivity. These discrepancies further reflect the variability in rsFC findings across studies, and the results from the current research contribute to this broader body of evidence.

Our results further revealed group differences in *changed* rsFC between the cerebellar network and regions in the temporal lobe. While mechanistic effects of bupropion should be interpreted with caution, this finding may be of interest given that the cerebellum, as well as cerebellar–temporal connections, have been implicated in MDD (Depping et al. 2018; Liu et al. 2012). Furthermore, the cerebellum could play an important role in modulating dopaminergic activity. For example, neuropathological changes in the cerebellum commonly observed in psychiatric and cognitive disorders may lead to dysfunctional cerebellar-mPFC circuitry, resulting in abnormal dopaminergic activity in the mPFC, with glutamate playing a key modulatory role (Rogers et al. 2011). A recent 2024 study found that the cerebellum directly modulates basal ganglia dopamine levels through monosynaptic glutamatergic projections to the substantia nigra pars compacta, influencing movement initiation, vigor, and reward processing in mouse models (Washburn et al. 2024). While not a priori predicted results, our preliminary findings could potentially indicate modulation of the dopaminergic system extending beyond the striatum, potentially involving cerebellar pathways that influence broader neural networks that might be relevant to MDD. To our knowledge, there is limited evidence for other types of antidepressant actions on the cerebellar network that could indicate specific effects of bupropion.

In addition to these findings, we observed significant differences in insula connectivity when using the seed-based approach. The insular cortex is part of SN and is important for interoceptive awareness, emotional regulation, and the integration of sensory information (Lynch et al. 2024; Zhang et al. 2024). Moreover, the insula and salience network have reciprocal connections with the dopaminergic midbrain related to reward processing and motivation (Lynch et al. 2024; Namkung et al. 2017; Peters et al. 2016; Seeley 2019). With regard to depression, a recent study showed that the frontostriatal salience network is expanded nearly twofold in the cortex of individuals with depression (Lynch et al. 2024), and meta-analyses have identified differences in both FC and brain structure within the insula in patients with depression (Fitzgerald et al. 2008; Peng et al. 2016). In this study, group comparisons showed changed rsFC between the insula and regions in the sensorimotor and occipital cortex, as well as in the precuneus. The precuneus has received greater attention in recent time due to associations with a range of key functions, including self-reflection, visuospatial processing, and episodic memory retrieval. The precuneus has also been considered a central hub of both DMN and ECN (Dadario and Sughrue 2023). Therefore, it is possible that bupropion exerts part of its clinical effects via these connections. Again, these potential mechanistic interpretations should be made cautiously, given the study design and lack of correlation with a reduction in symptom severity.

Several limitations should be considered when interpreting the findings of this study. The study design meant that all participants who received bupropion were depressed, whereas all participants who received no drug treatment were not depressed, thereby introducing a confounding variable into the study. This makes it challenging to determine whether group-by-visit interactions result from drug effects or from differences between the depressed and healthy groups. Moreover, the study did not include a placebo control group due to ethical concerns about leaving the patient group untreated for a long period with a placebo instead of receiving active medication. The controls were not given any medication, placebo, or other interventions during this 6-week period to allow assessment of repeated testing. A placebo control group could improve our interpretation of whether the observed effects are due to bupropion itself or to other factors, such as placebo effects, or other biases and natural fluctuations. Without this consideration, it is challenging to separate the true effects of bupropion from confounding factors. Another central limitation is that our study was only powered to detect medium to large effect sizes (d = 0.63 based on a reversed power calculation for the baseline comparison). Even though our study is not atypical in terms of sample size (see for example Zhang et al. 2025 and Saberi et al. 2024), even larger sample sizes (i.e., > 80 participants may be necessary to detect smaller differences between patient groups and controls, as well as effects of bupropion. Moreover, while we applied multiple comparison correction across voxels within each network and seed, results did not survive applying Bonferroni or FDR correction across the number of networks and seeds regions investigated. There are also limitations related to the lack of field map measures and corrections of the fMRI data. Current results should therefore be considered exploratory.

## Conclusion

Given that bupropion might be a preferable treatment option for certain individuals or subtypes of MDD, studies on its effects are warranted. While results are preliminary and do not support strong effects of bupropion, we found specific effects on connectivity within the cerebellar network, as well as on insula connections, which are both known to modulate striatal dopamine. Future studies should seek to replicate these effects. This study is the first to explore how bupropion treatment may change rsFC in patients with MDD, and our results could serve as important targets for future research.

## Supplementary Information

Below is the link to the electronic supplementary material.

Supplementary Material 1

## Data Availability

The data that support the findings of this study are not openly available for public use.
