# Supplementary material for: Treatment response to bupropion: an investigation of changes in resting-state functional connectivity in patients with major depressive disorder
Source: Psychopharmacology (Berl). 2025 Dec 23;243(10):2399–410. doi: 10.1007/s00213-025-06993-7 (PMC13683485; doi:10.1007/s00213-025-06993-7)
Supplement: Supplementary file 1 [file 213_2025_6993_MOESM1_ESM.docx]

**Supplementary Materials**

**Supplementary Table S1.**

Nonsignificant Differences at Baseline within Resting-State Networks between Patients and Controls

| **Network** | **Contrast** | ***p*-value** |
| --- | --- | --- |
| Visual | C > P | .773 |
|  | P > C | .112 |
| DMN/Striatum | C > P | .903 |
|  | P > C | .193 |
| Dorsal Attention/Visual Lateral | C > P | .591 |
|  | P > C | .490 |
| Executive Control | C > P | .325 |
|  | P > C | .630 |
| Sensory Motor/ACC and Frontal Gyrus | C > P | .098 |
|  | P > C | .674 |
| Salience (Part of) | C > P | .731 |
|  | P > C | .602 |
| Sensory Motor/Auditory | C > P | .957 |
|  | P > C | .394 |
| Frontoparietal (Left) | C > P | .648 |
|  | P > C | .193 |
| Frontoparietal (Right) | C > P | .300 |
|  | P > C | .649 |
| DMN/Frontoparietal/Salience | C > P | .190 |
|  | P > C | .534 |
| DMN (Part of) | C > P | .387 |
|  | P > C | .255 |
| Cerebellar | C > P | .122 |
|  | P > C | .243 |

*Note.* Nonsignificant differences in functional connections (temporal correlations/cohesion) within resting-state networks between patients and controls at baseline. P: Patients; C: Controls.

**Supplementary Table S2.**

Nonsignificant Differences in Connectivity in Seed Regions between Patients and Controls at Baseline

| **Seed** | **Contrast** | ***p*-value** |
| --- | --- | --- |
| Anterior DMN | C > P | .515 |
|  | P > C | .549 |
| Insula | C > P | .147 |
|  | P > C | .961 |
| Precuneus | C > P | .345 |
|  | P > C | .738 |
| Midbrain Striatum (Bilateral) | C > P | .258 |
|  | P > C | .284 |
| Midbrain Striatum (Left) | C > P | .281 |
|  | P > C | .354 |
| Midbrain Striatum (Right) | C > P | .289 |
|  | P > C | .224 |

*Note.* Nonsignificant differences in functional connections (temporal correlations/cohesion) in seed regions between patients and controls at baseline. P: Patients; C: Controls; DMN: Default Mode Network.

**Supplementary Table S3.**

Nonsignificant Changes between Posttreatment and Baseline within Resting-State Networks: Patients vs. Controls

| **Network** | **Contrast** | ***p*-value** |
| --- | --- | --- |
| Visual | C > P | .602 |
|  | P > C | .566 |
|  | Patients only | .560 |
| DMN/Striatum | C > P | .537 |
|  | P > C | .886 |
|  | Patients only | .483 |
| Dorsal Attention/Visual Lateral | C > P | .785 |
|  | Patients only | .684 |
| Executive Control | C > P | .579 |
|  | P > C | .284 |
|  | Patients only | .455 |
| Sensory Motor/ACC and Frontal Gyrus | C > P | .553 |
|  | P > C | .175 |
|  | Patients only | .522 |
| Salience (Part of) | C > P | .134 |
|  | P > C | .696 |
|  | Patients only | .793 |
| Sensory Motor/Auditory | C > P | .355 |
|  | P > C | .206 |
|  | Patients only | .440 |
| Frontoparietal (Left) | P > C | .618 |
|  | Patients only | .434 |
| Frontoparietal (Right) | C > P | .177 |
|  | P > C | .897 |
|  | Patients only | .814 |
| DMN/Frontoparietal/Salience | C > P | .879 |
|  | P > C | .188 |
|  | Patients only | .507 |
| DMN (Part of) | C > P | .094 |
|  | P > C | .232 |
|  | Patients only | .903 |
| Cerebellar | P > C | .519 |
|  | Patients only | .423 |

*Note.* Nonsignificant changes in resting-state FC networks between patients and controls. The table represents contrasts from the main analysis and the sensitivity analysis (patients only). P: Patients; C: Controls.

**Supplementary Table S4.**

Nonsignificant Changes between Posttreatment and Baseline in Seed Regions: Patients vs. Controls

| **Seed** | **Contrast** | ***p*-value** |
| --- | --- | --- |
| Anterior DMN | C > P | .537 |
|  | P > C | .144 |
|  | Patients only | .193 |
| Insula | C > P | .832 |
|  | Patients only | .245 |
| Precuneus | C > P | .858 |
|  | P > C | .290 |
|  | Patients only | .094 |
| Midbrain Striatum (Bilateral) | C > P | .227 |
|  | P > C | .234 |
|  | Patients only | .846 |
| Midbrain Striatum (Left) | C > P | .159 |
|  | P > C | .125 |
|  | Patients only | .545 |
| Midbrain Striatum (Right) | C > P | .170 |
|  | P > C | .635 |
|  | Patients only | .979 |

*Note.* Nonsignificant changes in functional connections (temporal correlations/cohesion) in seed regions between the patients and the controls. P: Patients; C: Controls, DMN: Default Mode Network.
